# Acute Zonal Occult Outer Retinopathy with Atypical Findings

**DOI:** 10.1155/2014/290696

**Published:** 2014-07-21

**Authors:** Dimitrios Karagiannis, Georgios A. Kontadakis, Artemios S. Kandarakis, Nikolaos Markomichelakis, Ilias Georgalas, Efstratios A. Parikakis, Stamatina A. Kabanarou

**Affiliations:** ^1^Ophthalmiatreio Eye Hospital of Athens, Eleftheriou Venizelou 26, 106 72 Athens, Greece; ^2^Department of Ophthalmology, General Hospital of Athens, Leoforos Mesogion 154, 115 27 Athens, Greece; ^3^Ophthalmology Department, Red Cross Hospital, Erythrou Stavrou 1, 115 26 Athens, Greece

## Abstract

*Background*. To report a case of acute zonal occult outer retinopathy (AZOOR) with atypical electrophysiology findings. *Case Presentation*. A 23-year-old-female presented with visual acuity deterioration in her right eye accompanied by photopsia bilaterally. Corrected distance visual acuity at presentation was 20/50 in the right eye and 20/20 in the left eye. Fundus examination was unremarkable. Visual field (VF) testing revealed a large scotoma. Pattern and full-field electroretinograms (PERG and ERG) revealed macular involvement associated with generalized retinal dysfunction. Electrooculogram (EOG) light rise and the Arden ratio were within normal limits bilaterally. The patient was diagnosed with AZOOR due to clinical findings, visual field defect, and ERG findings. *Conclusion*. This is a case of AZOOR with characteristic VF defects and clinical symptoms presenting with atypical EOG findings.

## 1. Introduction

Acute zonal occult outer retinopathy (AZOOR) is a rare retinal disorder first described in the literature by Gass in 1992 [1, 2]. Patients with AZOOR typically present with photopsia and scotomas. Fundoscopy in patients at initial presentation may be normal and diagnosis is confirmed with the abnormal findings in electrophysiology [1, 2].

According to Gass, AZOOR belongs to “white dot” syndromes, which is a wide spectrum of idiopathic inflammatory retinal disorders [1, 2]. Acute zonal occult outer retinopathy affects mostly women, with an average age at presentation of all patients described in the literature about 37 years. Typical characteristic of the disease is the presentation with vision deterioration in an area of the visual field accompanied by photopsia. Most of the patients present with unilateral disease, but involvement of the contralateral eye ultimately appears in the majority of the cases according to the literature [1].

In electrophysiology, most of the patients described in the literature have abnormal electroretinogram (ERG) in at least one eye (the most severely affected in asymmetric disease) [1, 2]. The electrooculogram (EOG) was also tested in many of the cases described in the literature, and so far all of them had abnormal results [1, 3].

The purpose of our study is to describe a case of AZOOR with typical clinical findings and abnormal ERG that presented with normal EOG, in contrast to the up till now published reports.

## 2. Case Presentation

**Figure 1:**
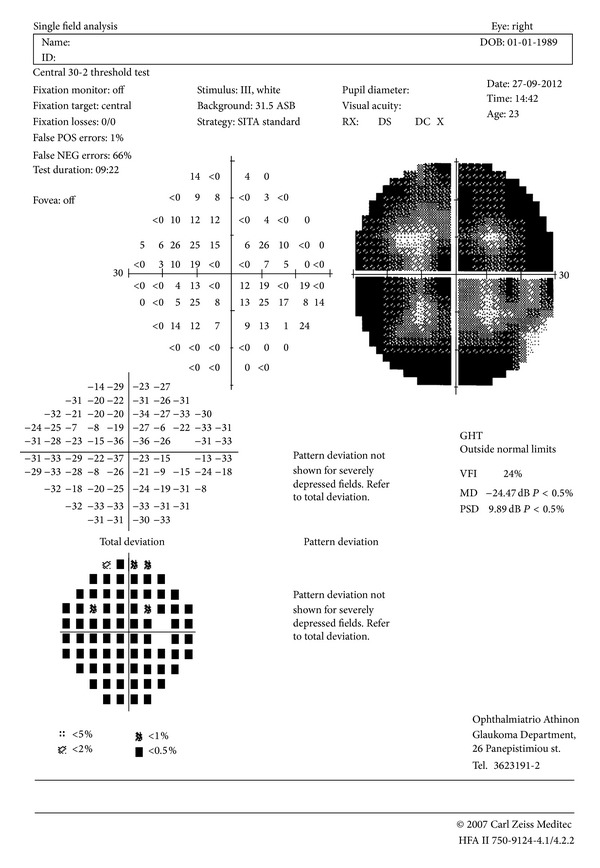
Visual field of the patient's right eye demonstrating a generalized depression.

A 23-year-old-female patient presented in the Outpatients Department of the Ophthalmiatreio Eye Hospital of Athens due to vision deterioration in her right eye and photopsias bilaterally. The patient underwent complete evaluation with detailed ophthalmic and systemic history, assessment of corrected distance visual acuity (CDVA), and slit lamp inspection of anterior and posterior segments. The patients' CDVA was 20/50 in the right eye and 20/20 in the left eye. Intraocular pressure was 14 mmHg in the right eye and 15 mmHg in the left eye. The patient had no history of other ophthalmic or systemic diseases. Anterior and posterior segments examination was unremarkable. The patient underwent visual field testing that revealed a large central scotoma in the right eye and was normal in the left eye (Figure 1).

**Figure 2:**
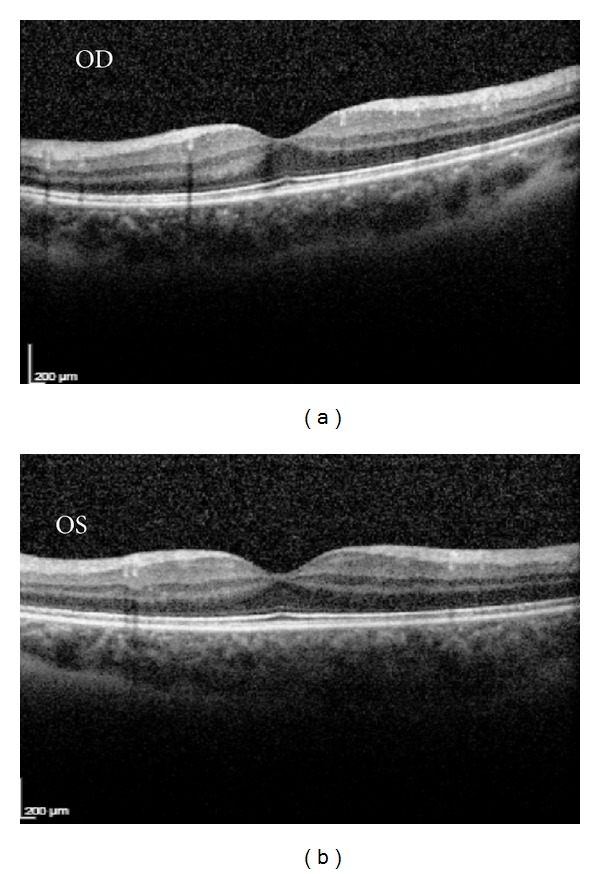
Optical coherence tomography of the macula without any abnormal findings.

Fluorescein angiography and indocyanine green angiography were unremarkable bilaterally. Optical coherence tomography (OCT) (Spectralis SD-OCT, Heidelberg Engineering, Inc., Germany) also revealed no macular pathology (Figure 2).

Additionally, the patient underwent PERG and ERG electroretinograms according to the protocols recommended by the International Society for Clinical Electrophysiology of Vision (ISCEV) [4, 5]. Stimulus 0.6 log units stronger than the ISCEV maximum standard flash was also used better to demonstrate the dark adapted a-wave (DA 11.0). Visual evoked potentials to a pattern stimulus (PVEP) and (EOG) were also recorded according to ISCEV standards [6, 7].

**Figure 3:**
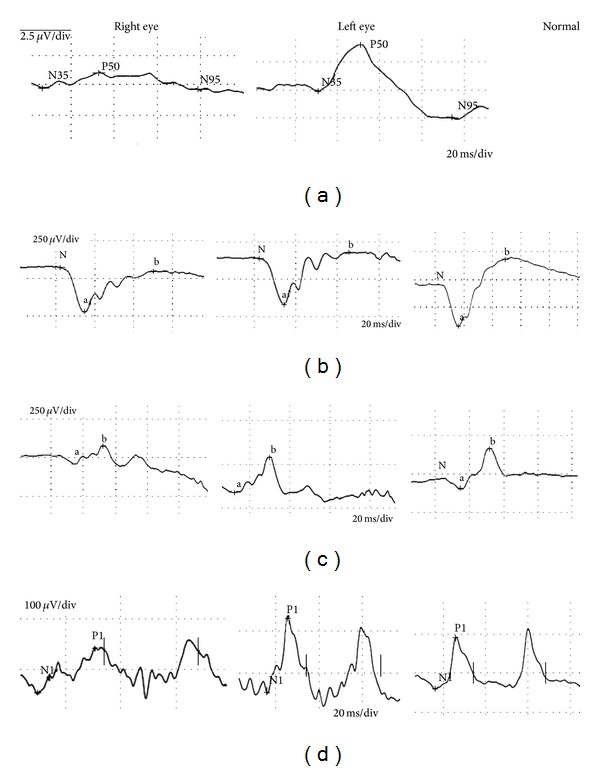
(a) Pattern electroretinogram (PERG) showing reduction of the P50 amplitude in the right eye. (b) Bright flash dark adapted ERGs (DA 11.0) demonstrating a “negative” ERG in the right eye, as b-wave is of a lower amplitude than a-wave. (c) Photopic single flash ERGs (LA 3.0) responses were subnormal in the right eye. (d) Light adapted 30 Hz flicker ERG (LA 30 Hz) demonstrating implicit time delay in the right eye.

According to the results, PERG P50 amplitude as an index of macular function was reduced in the right eye but was within normal limits in the left eye indicating right eye macular dysfunction (Figure 3(a)). The PVEP P_100_ component was of reduced amplitude and normal peak time in the right eye (possibly secondary to macular dysfunction rather than reflecting primary optic nerve disease) while responses in the left eye were recorded within normal range.

**Figure 4:**
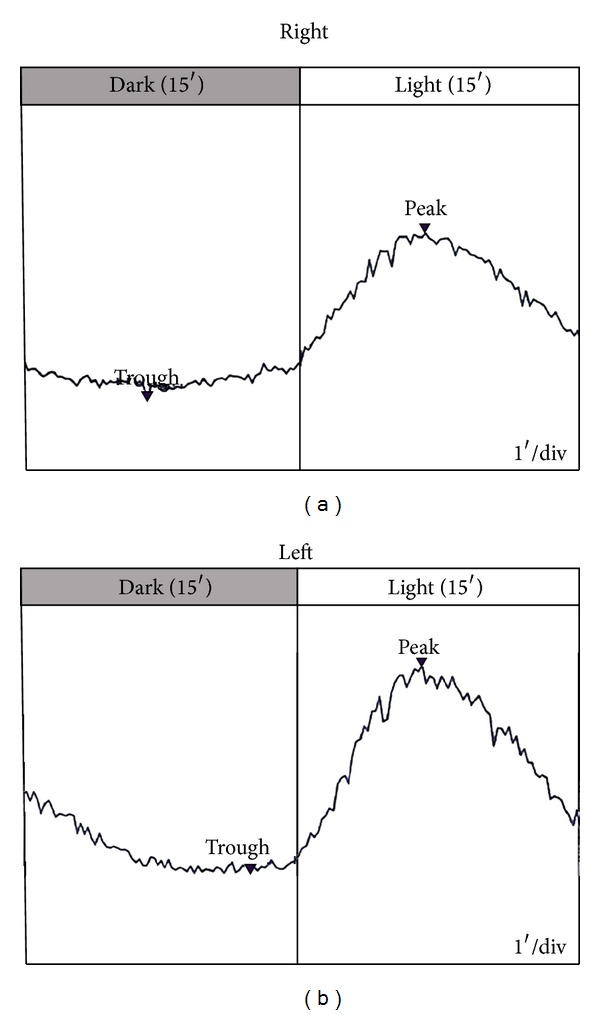
EOG recordings demonstrating a normal light rise and Arden ratio bilaterally.

Rod specific ERG (DA 0.01) was of reduced amplitude in the right eye only. Bright flash dark adapted ERGs (DA 11.0) were of normal a-wave amplitude bilaterally, while b-wave was of reduced amplitude in the right eye and subnormal in the left eye, indicating a “negative ERG” in the right eye (a-wave amplitudes: 302 *μ*V and 305 *μ*V in the right and left eye, resp.; b-wave amplitudes: 276 *μ*V and 345 *μ*V in the right and left eye, resp.) (Figure 3(b)). Photopic single flash ERGs (LA 3.0) responses were subnormal in the right eye only (a-wave amplitudes: 25 *μ*V and 38.5 *μ*V in the right and left eye, resp.; b-wave amplitudes: 115 *μ*V and 200 *μ*V in the right and left eye (Figure 3(c)). Light adapted 30 Hz flicker ERG (LA 30 Hz) was delayed in the right eye but within normal limits in the left eye (implicit time of 31 msec and 26 msec in the right and left eyes, resp.) (Figure 3(d)). EOG light rise and the Arden ratio were within normal limits bilaterally (Arden ratio: 4.2 and 3.9 in the right and left eyes, resp.) (Figure 4).

The electrophysiological findings thus indicated generalized retinal dysfunction (affecting both cone and rod systems and inner retina) associated with macular involvement in the right eye only. The patient was diagnosed with AZOOR due to typical clinical, visual field and ERG findings despite the normal EOG findings.

**Figure 5:**
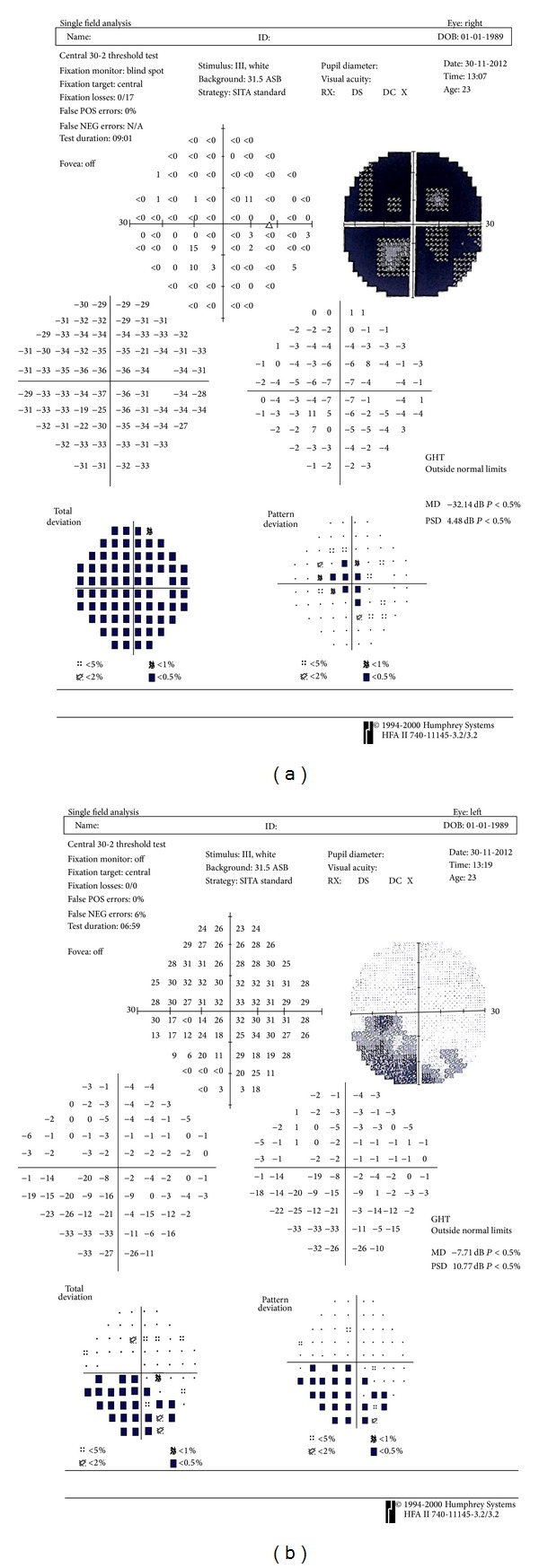
Visual field of right eye (a) and left eye (b) demonstrating deterioration of the right eye and involvement of the left.

Two months after first visit, patient evaluation was repeated. Vision in her right eye had deteriorated to 20/63, while in the left eye it was stable at 20/20. Visual field testing was also repeated and revealed further depression of sensitivity in the right eye and involvement of the left eye (Figure 5). The patient was followed up for 8 additional months and her symptoms and clinical findings were stabilized bilaterally. In the course of follow-up she also underwent magnetic resonance imaging of head and abdomen without findings.

## 3. Discussion

Acute zonal occult outer retinopathy is a rare disease of unknown etiology affecting mostly young female adults [1]. There are only a few studies and case reports in the literature of this condition describing electroretinography as the critical test for confirmation of diagnosis in addition to the clinical findings and the visual field defect [1–3]. To the best of our knowledge, this case is the first described in the literature with nontypical electrophysiology due to the normal findings in EOG.

Our case presented with vision deterioration in one eye and photopsias bilaterally. Photopsias are described as a symptom at presentation in the vast majority of the cases of AZOOR [1]. Visual acuity is not always affected, since most of eyes retain a visual acuity of 20/20. Still, there are a percentage of patients with impaired visual acuity. In a series of patients presented by Gass et al. CDVA was 20/40 or more in 76% of patients at presentation and final visual acuity was 20/40 or more in 68% of patients [2]. Slit lamp biomicroscopy and fundus imaging examination (FA and ICG) were unremarkable in our case, which according to the literature is a common condition in diagnosed AZOOR cases, since 50% of published cases present with no related findings in FA [1]. Optical coherence tomography (OCT) of the macula was normal. According to the literature, OCT may show disruptions in the inner segment/outer segment junction line and in the outer segment tip lines of the photoreceptors, especially in the late phase of the disease [8].

Visual field defect is also one of the main findings in cases of AZOOR. Several types of visual field defects have been observed, such as blind spot enlargement, constriction of the peripheral visual field, and central scotomas [1]. In our case the patient presented with a full scotoma of the central visual field in the initially affected eye which was not related to any other optic nerve pathology. The course of our patient (deterioration of the most affected eye in the first months and involvement of the contralateral eye) was also typical of AZOOR.

Electrophysiology is the critical testing for confirmation of diagnosis in cases of AZOOR [1–3]. Gass et al. [2] reported abnormal findings in ERG of all 51 patients in their series, and most of the published cases in the literature report abnormal ERGs [1]. Common findings are depressed scotopic and/or photopic amplitudes in one eye (the most severely affected) or both eyes [2]. A negative amplitude is not common but was also reported in the literature in one case by Gass et al. [2] and in one case by Piao et al., where dysfunction of the inner retina, as well as the outer retina, was proposed in such cases [9]. Our patient also presented with a “negative” ERG in his right eye. Macular involvement was also demonstrated in that eye.

In a retrospective series of 28 patients Francis et al. [3] identified a pattern of electrophysiological anomalies in AZOOR cases where all affected eyes demonstrated delayed implicit time of the 30 Hz cone flicker ERG. Our case also displayed delayed implicit time in his right eye which is in accordance with these criteria. In the aforementioned case series [3] all of the patients had a marked reduction in the EOG light rise, indicating a generalized dysfunction of the retinal pigment epithelium. Accordingly, case reports in the published literature that include EOG testing of the affected eyes report abnormal results of EOG [1]. In our case EOG light rise and the Arden ratio were within normal limits bilaterally, despite the fact that the rest of the test results were suggestive of AZOOR.

In all reported cases and series of patients in the literature there is no definite description of the disease. Even the pathophysiology of AZOOR is not clear since it is either considered part of the white dot disease spectrum or triggered by inflammatory disorders such as punctuate inner choroiditis and multifocal inner choroiditis [3]. Differential diagnosis of cases of AZOOR with such findings at presentation as in our case includes other conditions such as cancer associated retinopathy and melanoma associated retinopathy [1]. Our patient had clear medical history and was referred to for complete evaluation for possible occult malignancy including clinical examination, blood testing, and chest and abdominal MRI which were not suggestive of such condition.

## 4. Conclusion

In conclusion, our patients had typical clinical findings of AZOOR with photopsia and visual field defect. However, it represents an unusual case of AZOOR as she presented with a “negative” ERG, indicating an abnormal inner and outer retina dysfunction, and with normal EOG recordings. The latter may indicate that involvement of retinal pigment epithelium, as demonstrated by EOG abnormality, is not imperative in the course of AZOOR.
